# 752. Development of an Outpatient Infectious Disease Rotation for Internal Medicine Residents at a Rural Academic Medical Center

**DOI:** 10.1093/ofid/ofad500.813

**Published:** 2023-11-27

**Authors:** Nicole Bryan, Melanie A Fisher

**Affiliations:** West Virginia University, Morgantown, West Virginia; West Virginia University, Morgantown, West Virginia

## Abstract

**Background:**

Infectious disease (ID) training is essential to the education of internal medicine residents and has traditionally occurred on inpatient rotations focused on hospitalized patients. However, these rotations offer limited experience with infectious diseases managed in the outpatient setting. Furthermore, inpatient rotations expose residents to only one aspect of the multifaceted contributions ID specialists provide. Given the nationwide decline in ID providers, demonstrating the diverse career paths that can stem from ID training is a potential opportunity to attract trainees to this field. Developing interest in ID amongst residents is even more crucial in rural areas where the infectious disease provider shortage is severe. Here, we describe an ambulatory ID rotation for internal medicine residents at a rural academic medical center. We provide a model for a structured rotation with diverse learning opportunities for medical residents, and show the rotation has been received positively by those residents who have completed it to date.

**Methods:**

We established an outpatient ID rotation to offer as an elective for internal medicine residents. The rotation consisted of working one-on-one with ID faculty in a variety of settings in 2 or 4 week rotation blocks. A schedule was provided to each resident. Anonymous evaluations of the rotation were obtained upon completion of each block and aggregated.

**Results:**

There were a total of 11 internal medicine residents who participated in the ambulatory ID rotation from 7/1/2020 until 1/3/2023. Residents participated in a variety of clinical experiences, conferences, and didactics as summarized in Table 1. Each day of the rotation was divided into morning and afternoon clinics with conferences and didactics scheduled first thing in the morning or at noon. A sample rotation schedule is provided in Figure 1. Resident evaluations of the rotation were positive overall as summarized in Table 2.

**Figure:**
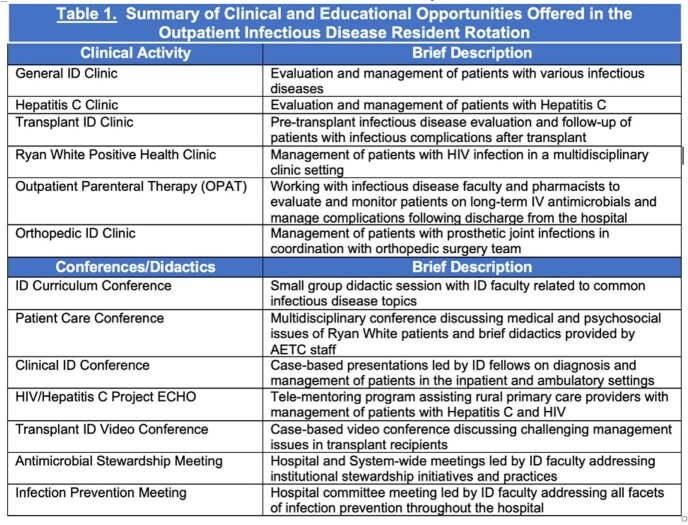

**Figure 1.**
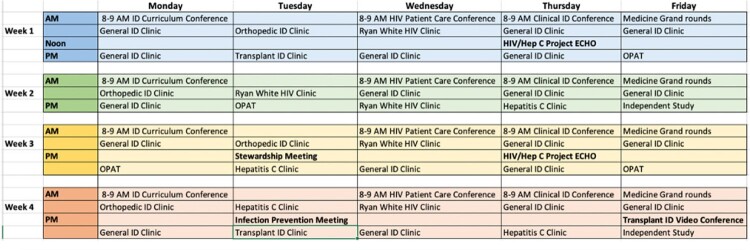
Example medical resident rotation schedule for outpatient ID elective rotation

**Figure:**
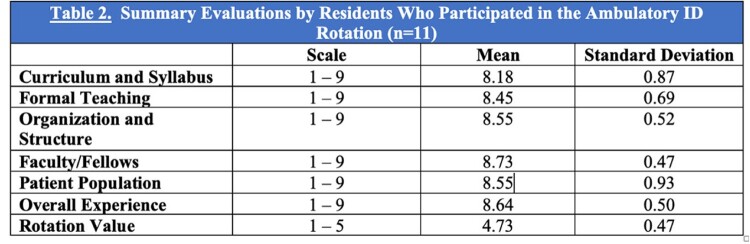

**Conclusion:**

We present a model for an ambulatory outpatient ID rotation consisting of a wide variety of educational and clinical opportunities that also provides a more comprehensive overview of different career paths within the ID specialty. Residents who have completed the rotation today have evaluated it favorably.

**Disclosures:**

**All Authors**: No reported disclosures

